# (8-Bromo-2-hydroxy-7-methoxy-1-naph­thyl)(4-chlorobenzoyl)methanone

**DOI:** 10.1107/S1600536810006185

**Published:** 2010-02-20

**Authors:** Ryosuke Mitsui, Kosuke Nakaema, Atsushi Nagasawa, Keiichi Noguchi, Noriyuki Yonezawa

**Affiliations:** a Department of Organic and Polymer Materials Chemistry, Tokyo University of Agriculture & Technology, 2-24-16 Naka-machi, Koganei, Tokyo 184-8588, Japan; b Instrumentation Analysis Center, Tokyo University of Agriculture & Technology, 2-24-16 Naka-machi, Koganei, Tokyo 184-8588, Japan

## Abstract

In the title compound, C_18_H_12_BrClO_3_, the naphthalene ring system and the benzene ring make a dihedral angle of 82.18 (9)°. The conformation around the central C=O group is such that the C=O bond vector forms a larger angle to the plane of the naphthalene ring system than to the plane of the benzene ring, *viz.* 60.91 (16)° *versus* 13.94 (16)°. In the crystal structure, two π–π inter­actions formed between the naphthalene ring systems [centroid–centroid distances of 3.8014 (13) and 3.9823 (13) Å] and inter­molecular O—H⋯O and C—H⋯O hydrogen bonds are present.

## Related literature

For the structures of closely related compounds, see: Mitsui, Nakaema, Noguchi, Okamoto & Yonezawa (2008 ▶); Mitsui, Nakaema, Noguchi & Yonezawa (2008 ▶); Mitsui *et al.* (2009 ▶).
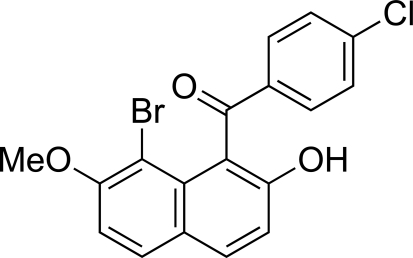


## Experimental

### 

#### Crystal data

C_18_H_12_BrClO_3_
*M* *_r_* = 391.64
Monoclinic, 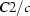

*a* = 23.1440 (4) Å
*b* = 7.61524 (14) Å
*c* = 20.2652 (4) Å
β = 112.733 (1)°
*V* = 3294.22 (10) Å^3^
*Z* = 8
Cu *K*α radiation
μ = 5.00 mm^−1^
*T* = 193 K
0.35 × 0.10 × 0.05 mm

#### Data collection

Rigaku R-AXIS RAPID diffractometer
Absorption correction: numerical (*NUMABS*; Higashi, 1999 ▶) *T*_min_ = 0.353, *T*_max_ = 0.779
12588 measured reflections
3004 independent reflections
2777 reflections with *I* > 2σ(*I*)
*R*_int_ = 0.032

#### Refinement

*R*[*F* ^2^ > 2σ(*F* ^2^)] = 0.028
*wR*(*F* ^2^) = 0.067
*S* = 1.30
3004 reflections
213 parameters
1 restraint
H atoms treated by a mixture of independent and constrained refinement
Δρ_max_ = 0.88 e Å^−3^
Δρ_min_ = −0.74 e Å^−3^

### 

Data collection: *PROCESS-AUTO* (Rigaku, 1998 ▶); cell refinement: *PROCESS-AUTO*; data reduction: *CrystalStructure* (Rigaku/MSC, 2004 ▶); program(s) used to solve structure: *SHELXS97* (Sheldrick, 2008 ▶); program(s) used to refine structure: *SHELXL97* (Sheldrick, 2008 ▶); molecular graphics: *ORTEPIII* (Burnett & Johnson, 1996 ▶); software used to prepare material for publication: *SHELXL97*.

## Supplementary Material

Crystal structure: contains datablocks fb2182o, New_Global_Publ_Block, I. DOI: 10.1107/S1600536810006185/is2524sup1.cif

Structure factors: contains datablocks I. DOI: 10.1107/S1600536810006185/is2524Isup2.hkl

Additional supplementary materials:  crystallographic information; 3D view; checkCIF report

## Figures and Tables

**Table 1:** Hydrogen-bond geometry (Å, °)

*D*—H⋯*A*	*D*—H	H⋯*A*	*D*⋯*A*	*D*—H⋯*A*
O2—H2*O*⋯O1^i^	0.81 (2)	1.93 (2)	2.728 (2)	172 (2)
C3—H3⋯O1^i^	0.95	2.58	3.205 (3)	124

Symmetry code: (i) 
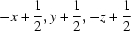
.

**Fig. 1.**
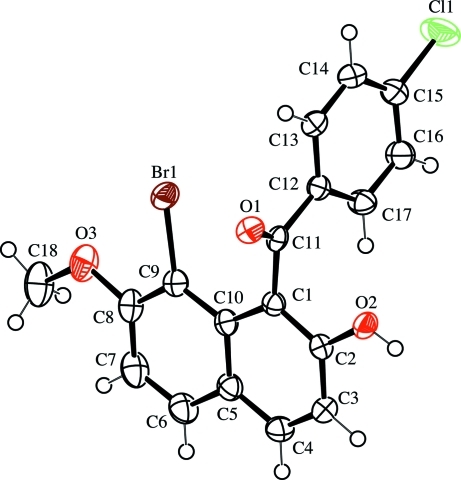
The molecular structure of compound (I), showing 50% probability displacement ellipsoids.

**Fig. 2.**
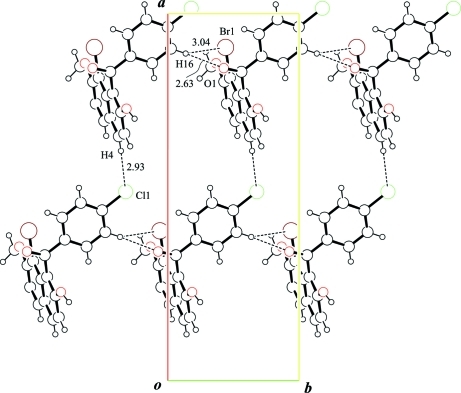
Partial crystal packing diagram of compound (I), viewed down the c axis. van der Waals interactions are shown as dashed lines.

**Fig. 3.**
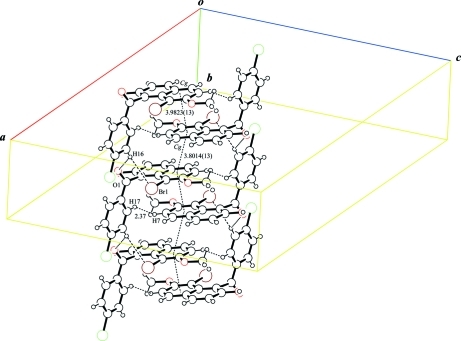
The arrangement of the molecules in the crystal structure, viewed in an oblique direction. van der Waals interactions and π– π interactions are shown as dashed lines.

**Fig. 4.**
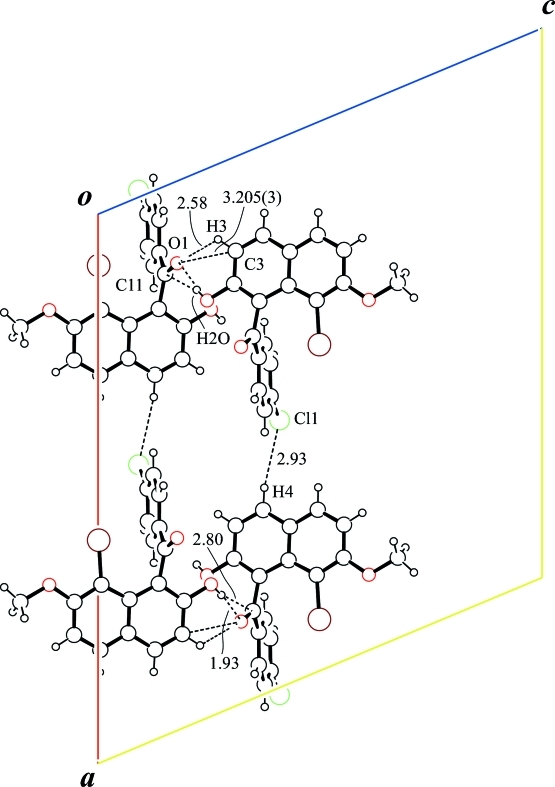
Partial crystal packing diagram of compound (I), viewed down the b axis. van der Waals interactions and intermolecular hydrogen bonds are shown as dashed lines.

## supplementary crystallographic information

### Comment

Recently, we have reported the crystal structures of 1-aroylated 2,7-dimethoxynaphthalene, 1-(4-chlorobenzoyl)-2,7-dimethoxynaphthalene (Mitsui, Nakaema, Noguchi, Okamoto & Yonezawa, 2008), (4-chlorophenyl)(2-hydroxy-7-methoxynaphthalen-1-yl)methanone (Mitsui, Nakaema, Noguchi & Yonezawa, 2008) and (4-chlorobenzoyl)(2-ethoxy-7-methoxynaphthalen-1-yl)methanone (Mitsui *et al.*, 2009). As a part of our ongoing studies on the synthesis and crystal structure analysis of aroylated naphthalene derivatives, we prepared and analysed the structure of crystal of 1-bromo-8-(4-chlorobenzoyl)-7-hydroxy-2-methoxynaphthalene, (I). The title compound was prepared by electrophilic aromatic bromination reaction of (4-chlorophenyl)(2-hydroxy-7-methoxynaphthalen-1-yl)methanone with bromine.

An *ORTEPIII* (Burnett & Johnson, 1996) plot of (I) is shown in Fig. 1. In the molecule of (I), the interplanar angle between the benzene ring (C12–C17) and the naphthalene ring (C1–C10) is 82.18 (9)°. The C═O bond vector and the least-squares plane of the benzene ring are relatively coplanar [13.94 (16)°]. By contrast, the C═O bond vector and the least-squares plane of the naphthalene ring are twisted [60.91 (15)°]. The conformation of these groups are similar to that of 1-(4-chlorobenzoyl)-2,7-dimethoxynaphthalene. Intriguingly, in the compound (I), there is no intramolecular hydrogen bond in contrast with (4-chlorophenyl)(2-hydroxy-7-methoxynaphthalen-1-yl)methanone. This is presumably caused by release of the large steric repulsion brought about by the benzene ring and the bromo group in the naphthalene ring of (I).

In the crystal structure, the molecular packing of (I) is stabilized by van der Waals interactions. The 4-chlorophenyl groups interact with the carbonyl groups [H16···O1 = 2.63 Å] and the bromo groups [H16···Br1 = 3.04 Å] along the *b* axis, and interact with the naphthalene rings [Cl1···H4 = 2.93 Å, H17···H7 = 2.37 Å] along the *a* axis (Figs. 2 and 3). The carbonyl groups interact with the hydroxy groups [C11···H2O = 2.80 Å] and the naphthalene rings [O1···C3 = 3.205 (3) Å] along the *b* axis (Fig. 4). Additionally, the naphthalene rings of neighbouring molecules are nearly parallel, and the π systems of the C5–C10 ring (with centroid *Cg*) in the naphthalene group are exactly parallel. The perpendicular distance between these aromatic rings is 3.4653 (9) and 3.6483 (9) Å. The centroid–centroid distance between the parallel aromatic rings is 3.8014 (13) and 3.9823 (13) Å, and the lateral offsets are 1.563 and 1.596 Å, indicating the presence of a π–π interaction (Fig. 3). Moreover, the crystal packing is stabilized by intermolecular hydrogen bonding between the carbonyl oxygen and hydrogen atom of the hydroxy group and naphthalene ring of the adjacent molecule *viz.* O2—H2O···O1 and C3—H3···O1 (Fig. 4 and Table 1).

### Experimental

To a solution of (4-chlorophenyl)(2-hydroxy-7-methoxynaphthalen-1-yl)methanone (313 mg, 1.00 mmol) in chloroform (5 ml) was added Br_2_ (161 mg, 1.01 mmol) drop-wise at 0 °C. The reaction mixture was stirred for 2 h at 0 °C, then poured into aqueous 2 *M* Na_2_S_2_O_3_ (10 ml). The precipitate was collected by vacuum filtration, and washed with several times with water. The crude material was purified by recrystallization from ethanol to give the title compound as a colorless blocks (m.p. 481.5–483.0 K, yield 333 mg, 85%).

Spectroscopic Data: ^1^H NMR (300 MHz, DMSO-*d*_6_) δ 10.26 (s, 1H), 7.98 (d, 1H), 7.92 (d, 1H), 7.74 (d, 2H), 7.53 (d, 2H), 7.33 (d, 1H), 7.11 (d, 1H), 3.90 (s, 3H); ^13^C NMR (75 MHz, DMSO-*d*_6_) δ 195.6, 155.1, 155.0, 138.3, 137.1, 131.8, 131.5, 130.2, 130.2, 128.5, 124.7, 118.4, 116.1, 110.1, 103.1, 56.7; IR (KBr): 3222, 1648, 1617, 1508, 1273, 1090; HRMS (*m*/*z*): [M + H]^+^ calcd for C_18_H_13_BrClO_3_, 390.9737; found, 390.9705.

Anal. Calcd for C_18_H_12_BrClO_3_: C 55.20, H 3.09. Found: C 55.04, H 2.97.

### Refinement

All the H atoms could be located in difference Fourier maps. The OH hydrogen atom was refined, with a bond restraint [O—H = 0.82 (2) Å], and with *U*_iso_(H) = 1.2*U*_eq_(O). The C-bound H atoms were subsequently refined as riding atoms, with C—H = 0.95 (aromatic) and 0.98 (methyl) Å, and with *U*_iso_(H) = 1.2*U*_eq_(C).

### Crystal data

**Table:**

C_18_H_12_BrClO_3_	*F*(000) = 1568
*M**_r_* = 391.64	*D*_x_ = 1.579 Mg m^−^^3^*D*_m_ = 1.57 Mg m^−^^3^*D*_m_ measured by picnomatar method
Monoclinic, *C*2/*c*	Melting point = 481.5–483.0 K
Hall symbol: -C 2yc	Cu *K*α radiation, λ = 1.54187 Å
*a* = 23.1440 (4) Å	Cell parameters from 11710 reflections
*b* = 7.61524 (14) Å	θ = 3.9–68.1°
*c* = 20.2652 (4) Å	µ = 5.00 mm^−^^1^
β = 112.733 (1)°	*T* = 193 K
*V* = 3294.22 (10) Å^3^	Block, colorless
*Z* = 8	0.35 × 0.10 × 0.05 mm

### Data collection

**Table:**

Rigaku R-AXIS RAPID diffractometer	3004 independent reflections
Radiation source: rotating anode	2777 reflections with *I* > 2σ(*I*)
graphite	*R*_int_ = 0.032
Detector resolution: 10.00 pixels mm^-1^	θ_max_ = 68.2°, θ_min_ = 4.1°
ω scans	*h* = −27→22
Absorption correction: numerical (*NUMABS*; Higashi, 1999)	*k* = −9→7
*T*_min_ = 0.353, *T*_max_ = 0.779	*l* = −24→24
12588 measured reflections	

### Refinement

**Table:**

Refinement on *F*^2^	Secondary atom site location: difference Fourier map
Least-squares matrix: full	Hydrogen site location: inferred from neighbouring sites
*R*[*F*^2^ > 2σ(*F*^2^)] = 0.028	H atoms treated by a mixture of independent and constrained refinement
*wR*(*F*^2^) = 0.067	*w* = 1/[σ^2^(*F*_o_^2^) + (0.0094*P*)^2^ + 5.4643*P*] where *P* = (*F*_o_^2^ + 2*F*_c_^2^)/3
*S* = 1.30	(Δ/σ)_max_ < 0.001
3004 reflections	Δρ_max_ = 0.88 e Å^−^^3^
213 parameters	Δρ_min_ = −0.74 e Å^−^^3^
1 restraint	Extinction correction: *SHELXL*, Fc^*^=kFc[1+0.001xFc^2^λ^3^/sin(2θ)]^-1/4^
Primary atom site location: structure-invariant direct methods	Extinction coefficient: 0.00022 (2)

### Special details

**Table:**

Geometry. All esds (except the esd in the dihedral angle between two l.s. planes) are estimated using the full covariance matrix. The cell esds are taken into account individually in the estimation of esds in distances, angles and torsion angles; correlations between esds in cell parameters are only used when they are defined by crystal symmetry. An approximate (isotropic) treatment of cell esds is used for estimating esds involving l.s. planes.
Refinement. Refinement of *F*^2^ against ALL reflections. The weighted *R*-factor wR and goodness of fit *S* are based on *F*^2^, conventional *R*-factors *R* are based on *F*, with *F* set to zero for negative *F*^2^. The threshold expression of *F*^2^ > σ(*F*^2^) is used only for calculating *R*-factors(gt) etc. and is not relevant to the choice of reflections for refinement. *R*-factors based on *F*^2^ are statistically about twice as large as those based on *F*, and *R*- factors based on ALL data will be even larger.

### Fractional atomic coordinates and isotropic or equivalent isotropic displacement parameters (Å^2^)

**Table:**

	*x*	*y*	*z*	*U*_iso_*/*U*_eq_	
Br1	0.093885 (11)	0.43969 (4)	0.001360 (12)	0.03654 (11)	
Cl1	−0.01318 (3)	1.17778 (10)	0.09112 (5)	0.0656 (3)	
O1	0.14955 (7)	0.4263 (2)	0.17786 (8)	0.0301 (4)	
O2	0.25985 (7)	0.6846 (2)	0.25441 (8)	0.0332 (4)	
H2O	0.2875 (10)	0.750 (3)	0.2780 (13)	0.040*	
O3	0.13753 (9)	0.3672 (3)	−0.10993 (8)	0.0441 (5)	
C1	0.21864 (10)	0.5799 (3)	0.13670 (11)	0.0245 (5)	
C2	0.26939 (10)	0.6442 (3)	0.19367 (11)	0.0274 (5)	
C3	0.32829 (11)	0.6678 (3)	0.18978 (13)	0.0333 (5)	
H3	0.3626	0.7123	0.2297	0.040*	
C4	0.33562 (11)	0.6261 (3)	0.12813 (13)	0.0362 (6)	
H4	0.3754	0.6421	0.1255	0.043*	
C5	0.28538 (11)	0.5595 (3)	0.06792 (13)	0.0313 (5)	
C6	0.29448 (13)	0.5147 (4)	0.00503 (14)	0.0388 (6)	
H6	0.3348	0.5300	0.0039	0.047*	
C7	0.24741 (13)	0.4504 (3)	−0.05395 (14)	0.0413 (6)	
H7	0.2551	0.4197	−0.0953	0.050*	
C8	0.18770 (12)	0.4300 (3)	−0.05318 (12)	0.0336 (5)	
C9	0.17682 (11)	0.4728 (3)	0.00745 (12)	0.0277 (5)	
C10	0.22513 (10)	0.5362 (3)	0.07094 (11)	0.0257 (5)	
C11	0.15967 (10)	0.5612 (3)	0.15115 (10)	0.0241 (5)	
C12	0.11712 (10)	0.7145 (3)	0.13796 (11)	0.0245 (5)	
C13	0.05714 (10)	0.6943 (3)	0.13793 (12)	0.0296 (5)	
H13	0.0438	0.5822	0.1471	0.036*	
C14	0.01708 (11)	0.8358 (3)	0.12465 (13)	0.0358 (6)	
H14	−0.0238	0.8222	0.1246	0.043*	
C15	0.03738 (11)	0.9985 (3)	0.11142 (14)	0.0374 (6)	
C16	0.09668 (12)	1.0226 (3)	0.11234 (14)	0.0382 (6)	
H16	0.1101	1.1355	0.1042	0.046*	
C17	0.13615 (11)	0.8797 (3)	0.12532 (13)	0.0319 (5)	
H17	0.1770	0.8946	0.1256	0.038*	
C18	0.14644 (16)	0.3312 (4)	−0.17507 (13)	0.0541 (8)	
H18A	0.1071	0.2885	−0.2115	0.065*	
H18B	0.1790	0.2416	−0.1660	0.065*	
H18C	0.1593	0.4390	−0.1921	0.065*	

### Atomic displacement parameters (Å^2^)

**Table:**

	*U*^11^	*U*^22^	*U*^33^	*U*^12^	*U*^13^	*U*^23^
Br1	0.03018 (15)	0.04598 (19)	0.02791 (14)	−0.00231 (11)	0.00512 (10)	−0.00651 (11)
Cl1	0.0416 (4)	0.0394 (4)	0.1165 (7)	0.0139 (3)	0.0313 (4)	0.0080 (4)
O1	0.0297 (8)	0.0266 (9)	0.0315 (8)	−0.0011 (7)	0.0090 (7)	0.0068 (7)
O2	0.0294 (9)	0.0405 (11)	0.0283 (8)	−0.0067 (7)	0.0094 (7)	−0.0108 (7)
O3	0.0567 (11)	0.0509 (12)	0.0239 (8)	−0.0086 (9)	0.0146 (8)	−0.0072 (8)
C1	0.0263 (11)	0.0210 (11)	0.0259 (10)	0.0015 (9)	0.0097 (9)	0.0016 (9)
C2	0.0283 (11)	0.0240 (12)	0.0300 (11)	0.0010 (9)	0.0113 (9)	−0.0020 (9)
C3	0.0281 (12)	0.0324 (13)	0.0367 (12)	−0.0027 (10)	0.0096 (10)	−0.0058 (11)
C4	0.0294 (12)	0.0360 (14)	0.0475 (14)	−0.0031 (11)	0.0196 (11)	−0.0036 (12)
C5	0.0351 (13)	0.0263 (13)	0.0370 (13)	−0.0013 (10)	0.0187 (10)	−0.0008 (10)
C6	0.0443 (14)	0.0386 (15)	0.0445 (14)	−0.0044 (12)	0.0291 (12)	−0.0027 (12)
C7	0.0609 (17)	0.0378 (15)	0.0360 (13)	−0.0052 (13)	0.0303 (13)	−0.0030 (11)
C8	0.0474 (15)	0.0271 (13)	0.0270 (11)	−0.0038 (11)	0.0153 (10)	−0.0006 (10)
C9	0.0326 (12)	0.0237 (12)	0.0271 (11)	0.0002 (10)	0.0117 (9)	0.0028 (9)
C10	0.0306 (11)	0.0199 (11)	0.0271 (11)	0.0001 (9)	0.0118 (9)	0.0020 (9)
C11	0.0259 (11)	0.0255 (12)	0.0171 (9)	−0.0026 (9)	0.0040 (8)	−0.0026 (8)
C12	0.0266 (11)	0.0256 (12)	0.0209 (10)	−0.0006 (9)	0.0088 (8)	−0.0003 (9)
C13	0.0281 (11)	0.0289 (13)	0.0312 (11)	−0.0046 (10)	0.0108 (9)	−0.0002 (10)
C14	0.0252 (12)	0.0376 (15)	0.0439 (14)	−0.0024 (11)	0.0126 (10)	−0.0035 (11)
C15	0.0301 (12)	0.0312 (14)	0.0483 (14)	0.0049 (11)	0.0121 (11)	−0.0020 (11)
C16	0.0357 (13)	0.0256 (13)	0.0533 (15)	−0.0002 (11)	0.0172 (12)	0.0037 (11)
C17	0.0282 (12)	0.0302 (13)	0.0399 (13)	−0.0004 (10)	0.0160 (10)	0.0036 (10)
C18	0.087 (2)	0.0512 (19)	0.0263 (13)	−0.0088 (17)	0.0243 (14)	−0.0067 (12)

### Geometric parameters (Å, °)

**Table:**

Br1—C9	1.892 (2)	C7—C8	1.397 (4)
Cl1—C15	1.741 (3)	C7—H7	0.9500
O1—C11	1.225 (3)	C8—C9	1.385 (3)
O2—C2	1.367 (3)	C9—C10	1.424 (3)
O2—H2O	0.808 (17)	C11—C12	1.484 (3)
O3—C8	1.366 (3)	C12—C17	1.389 (3)
O3—C18	1.439 (3)	C12—C13	1.396 (3)
C1—C2	1.380 (3)	C13—C14	1.379 (3)
C1—C10	1.437 (3)	C13—H13	0.9500
C1—C11	1.509 (3)	C14—C15	1.387 (4)
C2—C3	1.407 (3)	C14—H14	0.9500
C3—C4	1.362 (3)	C15—C16	1.378 (3)
C3—H3	0.9500	C16—C17	1.379 (3)
C4—C5	1.415 (3)	C16—H16	0.9500
C4—H4	0.9500	C17—H17	0.9500
C5—C6	1.411 (3)	C18—H18A	0.9800
C5—C10	1.430 (3)	C18—H18B	0.9800
C6—C7	1.360 (4)	C18—H18C	0.9800
C6—H6	0.9500		
			
C2—O2—H2O	108 (2)	C9—C10—C1	126.1 (2)
C8—O3—C18	117.5 (2)	C5—C10—C1	117.7 (2)
C2—C1—C10	119.9 (2)	O1—C11—C12	120.8 (2)
C2—C1—C11	114.35 (19)	O1—C11—C1	120.4 (2)
C10—C1—C11	125.79 (19)	C12—C11—C1	118.53 (19)
O2—C2—C1	116.9 (2)	C17—C12—C13	119.0 (2)
O2—C2—C3	121.3 (2)	C17—C12—C11	120.7 (2)
C1—C2—C3	121.9 (2)	C13—C12—C11	120.3 (2)
C4—C3—C2	119.2 (2)	C14—C13—C12	120.5 (2)
C4—C3—H3	120.4	C14—C13—H13	119.7
C2—C3—H3	120.4	C12—C13—H13	119.7
C3—C4—C5	121.5 (2)	C13—C14—C15	118.9 (2)
C3—C4—H4	119.2	C13—C14—H14	120.6
C5—C4—H4	119.2	C15—C14—H14	120.6
C6—C5—C4	120.4 (2)	C16—C15—C14	121.8 (2)
C6—C5—C10	119.8 (2)	C16—C15—Cl1	118.3 (2)
C4—C5—C10	119.8 (2)	C14—C15—Cl1	119.93 (19)
C7—C6—C5	122.2 (2)	C15—C16—C17	118.7 (2)
C7—C6—H6	118.9	C15—C16—H16	120.7
C5—C6—H6	118.9	C17—C16—H16	120.7
C6—C7—C8	119.4 (2)	C16—C17—C12	121.1 (2)
C6—C7—H7	120.3	C16—C17—H17	119.5
C8—C7—H7	120.3	C12—C17—H17	119.5
O3—C8—C9	116.2 (2)	O3—C18—H18A	109.5
O3—C8—C7	123.5 (2)	O3—C18—H18B	109.5
C9—C8—C7	120.2 (2)	H18A—C18—H18B	109.5
C8—C9—C10	122.2 (2)	O3—C18—H18C	109.5
C8—C9—Br1	116.00 (17)	H18A—C18—H18C	109.5
C10—C9—Br1	121.80 (17)	H18B—C18—H18C	109.5
C9—C10—C5	116.2 (2)		
			
C10—C1—C2—O2	179.4 (2)	C4—C5—C10—C9	178.4 (2)
C11—C1—C2—O2	−1.0 (3)	C6—C5—C10—C1	178.6 (2)
C10—C1—C2—C3	−0.5 (3)	C4—C5—C10—C1	−1.0 (3)
C11—C1—C2—C3	179.2 (2)	C2—C1—C10—C9	−178.4 (2)
O2—C2—C3—C4	−179.9 (2)	C11—C1—C10—C9	2.0 (4)
C1—C2—C3—C4	0.0 (4)	C2—C1—C10—C5	1.0 (3)
C2—C3—C4—C5	−0.1 (4)	C11—C1—C10—C5	−178.6 (2)
C3—C4—C5—C6	−179.0 (2)	C2—C1—C11—O1	−87.5 (3)
C3—C4—C5—C10	0.6 (4)	C10—C1—C11—O1	92.1 (3)
C4—C5—C6—C7	−180.0 (3)	C2—C1—C11—C12	87.5 (2)
C10—C5—C6—C7	0.4 (4)	C10—C1—C11—C12	−92.9 (3)
C5—C6—C7—C8	1.0 (4)	O1—C11—C12—C17	163.4 (2)
C18—O3—C8—C9	176.5 (2)	C1—C11—C12—C17	−11.6 (3)
C18—O3—C8—C7	−4.0 (4)	O1—C11—C12—C13	−16.8 (3)
C6—C7—C8—O3	179.7 (2)	C1—C11—C12—C13	168.26 (19)
C6—C7—C8—C9	−0.8 (4)	C17—C12—C13—C14	0.8 (3)
O3—C8—C9—C10	178.7 (2)	C11—C12—C13—C14	−179.0 (2)
C7—C8—C9—C10	−0.9 (4)	C12—C13—C14—C15	−0.1 (4)
O3—C8—C9—Br1	−0.6 (3)	C13—C14—C15—C16	−1.1 (4)
C7—C8—C9—Br1	179.81 (19)	C13—C14—C15—Cl1	177.56 (19)
C8—C9—C10—C5	2.2 (3)	C14—C15—C16—C17	1.4 (4)
Br1—C9—C10—C5	−178.52 (16)	Cl1—C15—C16—C17	−177.2 (2)
C8—C9—C10—C1	−178.4 (2)	C15—C16—C17—C12	−0.6 (4)
Br1—C9—C10—C1	0.9 (3)	C13—C12—C17—C16	−0.5 (3)
C6—C5—C10—C9	−2.0 (3)	C11—C12—C17—C16	179.4 (2)

### Hydrogen-bond geometry (Å, °)

**Table:**

*D*—H···*A*	*D*—H	H···*A*	*D*···*A*	*D*—H···*A*
O2—H2O···O1^i^	0.81 (2)	1.93 (2)	2.728 (2)	172 (2)
C3—H3···O1^i^	0.95	2.58	3.205 (3)	124

Symmetry codes: (i) −*x*+1/2, *y*+1/2, −*z*+1/2.
